# Air Pollution and Mortality in Chile: Susceptibility among the Elderly

**DOI:** 10.1289/ehp.9567

**Published:** 2007-01-08

**Authors:** Sabit Cakmak, Robert E. Dales, Claudia Blanco Vidal

**Affiliations:** 1 Department of Statistics, Health Canada, Ottawa, Ontario, Canada; 2 Department of Epidemiology, University of Ottawa, Ontario, Canada; 3 Area Descontaminacion Atmosferica, Comisión Nacional del Medio Ambiente (CONAMA), Metropolitana De Santiago, Chile

**Keywords:** air pollution, elderly, environment, epidemiology, mortality

## Abstract

**Objective:**

The estimated mortality rate associated with ambient air pollution based on general population studies may not be representative of the effects on certain subgroups. The objective of the present study was to determine the influence of relatively high concentrations of air pollution on mortality in a general population sample and in the very elderly.

**Study design:**

Daily time-series analyses tested the association between daily air pollution and daily mortality in seven Chilean urban centers during 1997–2003. Results were adjusted for day of the week and humidex.

**Results:**

Daily averaged particulate matter with aerodynamic matter < 10 μm (PM_10_) was 84.88 μg/m^3^, sulfur dioxide was 14.08ppb, and carbon monoxide was 1.29 ppb. The 1-hr maximum ozone was 100.13 ppb. The percentage increases in nonaccidental mortality associated with an increase in PM_10_ equivalent to its mean were 4.53 (t-ratio 1.52) for those < 65 years and 14.03 (3.87) for those > 85 years. Respective values were 4.96 (1.17) and 8.56 (2.02) for O_3_; 4.77 (2.50) and 7.92 (3.23) for SO_2_; and 4.10 (2.52) and 8.58 (4.45) for CO.

**Conclusion:**

Our results suggest that the very elderly are particularly susceptible to dying from air pollution. Concentrations deemed acceptable for the general population may not adequately protect the very elderly.

Acute exposure to ambient air pollution is associated with increased morbidity and mortality from cardiac and respiratory disease (Stieb et al. 2002). Investigating the possibility of susceptible subgroups is important to better understand the population health effects and guide air quality standards. The very young and very old are easily identifiable subgroups of interest. During the early periods of life, air pollution has been associated with preterm birth, intrauterine growth retardation, sudden infant death syndrome, and infant mortality (Dales et al. 2004; Ha et al. 2003; Liu et al. 2003; Ritz et al. 2002; Wang et al. 1997). Among the elderly, adverse effects of air pollution have been reported in those > 65 years of age (Schwartz 1994), but questions remain. Are these senior citizens more susceptible than those who are younger? What is the effect of air pollution at the further extremes of old age? To address these issues, we examined the association between pollution and mortality during the period 1997–2003 in seven Chilean cities.

## Methods

### Mortality and pollution data

We classified diseases according to the *International Classification of Disease, 9th Revision* (ICD-9; WHO 1975) codes. We obtained the daily number of deaths in metropolitan Santiago for all nonaccidental (ICD-9 codes < 800), cardiovascular (ICD-9 codes 390–459), and respiratory (ICD-9 codes 460–519) causes from the Instituto Nacional de Estadisticas, the official source of statistical data in Chile. We divided Santiago into urban centers based on proximity to seven air quality monitoring sites in the following municipalities: Las Condes, Cerrillos, El.Bosque, La Florida, Independencia, Santiago Centro, and Pudahuel. The period covered was from 1 January 1997 to 31 December 2003, totaling 2,557 days of observation in each urban center. Each urban center had daily (24-hr mean) measurements of ozone, sulfur dioxide, particulate matter < 10 μm in aerodynamic diameter (PM_10_), and carbon monoxide throughout the 7-year period of study. Daily average values for PM_10_, SO_2_, and CO and the daily 1-hr maximum concentrations of O_3_ were used in our analysis.

### Statistical methods

Daily variations in the number of deaths were related to daily variations in ambient concentrations of air pollutants by a random-effects regression model for count data (Burnett et al. 1995), which assumes a Poisson distribution of daily deaths. Here we assumed a linear association between air pollution and mortality on the logarithmic scale, with the association varying at random between cities. Mortality, air pollution, and weather can vary seasonally and are subject to longer-term time trends. Thus any examination of the association between short-term variations in air pollution and mortality must adjust for temporal trends in mortality and weather factors (Lumley and Sheppard 2003; Ramsay et al. 2003; Samet et al. 2003) and may perform better in this regard than the nonparametric model approaches used in previous studies (Cifuentes et al. 2000).

For each urban center separately, we fit natural spline functions to day of study with one knot for each of 15, 30, 60, 90, 120, 180, and 365 days of observation. We then selected a model with the number of knots that either minimized the Akaike information criterion (AIC) (Akaike 1973), a measure of model prediction, or maximized the evidence that the model residuals did not display any type of structure, including serial correlation, using Bartlett’s test (Priestly 1981). We also plotted model residuals against time and found neither a significant pattern nor correlation. Given our optimal model for time, we then selected our best weather predictor of mortality based on the humidex reading, a measure of the combined effect of temperature and humidity. We considered humidex readings on the day of death and the day before death and accounted for potential nonlinear associations with mortality by using natural spline functions. We examined linear weather models and models with up to 4 knots. The model that minimized the AIC was selected as the optimal weather model for each urban center separately. Indicator functions for day of the week were also included to account for any possible differences in mortality rates among the days of the week.

We then included location-specific time and weather models with air pollution in the regression model. Lagging times of 0–5 days were examined for the air pollutants. We also used unconstrained distributed lags as described by Schwartz (2000). The urban center–specific regression parameter estimates of the pollution–mortality association were then pooled among locations using random-effects models. The pooled estimates of the association between pollution and mortality, estimation of uncertainty, and estimate of variability between urban centers were determined by restricted maximum-likelihood methods (Lindstrom and Bates 1990).

The percentage change in mortality associated with changes in the population-weighted pollutant concentrations, equivalent in magnitude to their mean values, are reported in addition to the ratio of the estimate to its standard error (t-ratio). The association between ambient concentrations of air pollutants and mortality was determined for nonaccidental, cardiovascular, and respiratory causes of death, age at death, season, and adjustment for other air pollutants.

## Results

Population sizes ranged from 501,000 in the urban center represented by Las Condes to 1.34 million in La Florida, with a total of 5.37 million people in the study based on the 1996 census (Table 1). The death rate varied from 1.03 per 100,000 in Las Condes to 1.97 in Independencia. Total daily deaths averaged 69.69, of which 19.21 were cardiac and 8.38 were respiratory. PM_10_ concentrations ranged from 65.02 μg/m^3^ in Las Condes to 91.38 μg/m^3^ in Pudahuel, with a population-weighted average of 84.88 μg/m^3^. O_3_ concentrations ranged from 84.94 ppb in Pudahuel to 135.52 ppb in Las Condes, with a population-weighted average of 100.13 ppb. SO_2_ concentrations ranged from 9.12 ppb in Las Condes to 34.06 in Independencia, with a pooled average of 14.08 ppb; and CO concentrations ranged from 0.92 ppm in Las Condes and to 1.48 ppm in Santiago Centro, with a pooled average of 1.29 ppm.

Pairwise Pearson correlations between pollutants were variable between centers. Correlations between SO_2_ and CO and PM_10_ were always positive, whereas O_3_ was negatively correlated with SO_2_ and CO in some cities (Table 2). Correlations between PM_10_ and O_3_ and other pollutants varied from negative to positive, −0.55 to 0.82.

The number of knots used for each of the 28 location–pollutant associations was 14, except for associations between O_3_ and La Florida, Independencia, and Santiago Centro, for which the number of knots was 42, 84, and 84, respectively. The association between air pollutants and mortality was tested using lags of 0–5 days. In single-pollutant models using the best single-day lag, by urban center, each of the individual pollutants was significantly associated with an increased daily nonaccidental mortality evidenced by a t-ratio > 2 (Table 3). There were only two exceptions: PM_10_ in Las Condes and O_3_ in Independencia. Each of the associations were positive and effect sizes varied by < 3-fold between cities.

Averaged over urban centers, the mean single-day lags providing the greatest association were 2 days for O_3_, 1 day for SO_2_, 1 day for CO, and same day (unlagged) for PM_10_. With these lags in a single-pollutant model, the percent change in mortality attributable to a change in daily pollutants equivalent in magnitude to their mean ranged from 5.64% for O_3_ to 8.54% for PM_10_, with t-ratios > 2 (Table 4). These effects sizes diminished somewhat but all remained significant after adjustment for the other pollutants.

We examined the mortality effect of air pollution by lag structure, age, cause of death, and season (Table 5). The association between nonaccidental mortality and air pollutants was not significantly different when unconstrained distributed lags were substituted for single-day lags. The distributed but not the single-day effects of air pollution were observed to have an approximate 2-fold greater effect on respiratory than on cardiac deaths (*t*-test, *p* < 0.05 for PM_10_, SO_2_, and CO). Both single-day and unconstrained effects were consistent with an increased susceptibility of dying from air pollution among the elderly. Considering the distributed effects, the percent change in mortality was 2- to 3-fold greater for those at least 85 years of age compared to those < 65 years of age (*t*-test, *p* < 0.05 for PM_10_, SO_2_, and CO). For single-day lags, age- group differences varied from 3-fold for PM_10_ to a 57% increase for O_3_ (*t*-test, *p* < 0.05 for PM_10_). Larger risks were observed in the colder months of April–September compared to the warmer period of October–March for PM_10_ (*t*-test, *p* < 0.05).

## Discussion

Kavouras et al. (2001) observed that PM_10_ concentrations in 1998 in several Chilean cities were high by American and European standards. Our data are similar, with an annual average in metropolitan Santiago of 85 μg/m^3^ from 1997 through 2003. We did not have concentrations of fine particles < 2.5 μm in diameter (PM_2.5_) for all centers studied, but expect it would be associated with mortality. In the four where it was available, the annual average ranged from 26 to 35 μg/m^3^ and the correlations with PM_10_ varied from 0.78 to 0.90. Ilabaca et al. (1999) also demonstrated a strong day-to-day correlation between PM_10_ and PM_2.5_ between February 1995 and August 1996, with higher median values for both during the colder season. Jorquera et al. (2004) reported that because of geography and climatic conditions, Santiago has a higher ratio of estimated PM_10_ emissions (tons per year) to annual mean PM_10_ (micrograms per cubic meter) than Mexico City, Buenos Aires, and São Paulo. A low-lying inversion layer in the winter reduces dispersion of pollutants. Although the present study is based on outdoor-area monitoring, it probably reflects personal exposure. During the winters of 1988 and 1989, Rojas-Bracho et al. (2002) carried out an exposure study of Santiago children 10–12 years of age; personal, indoor, and outdoor PM_2.5_ concentrations were all within 5% at 69.5, 68.5, and 68.1 μg/m^3^, respectively. Even if the mean outdoor and indoor values are different, our results would be valid as long as the exposures changed in the same direction. The present daily time-series analysis examines the effects of day-to-day differences in air pollution, not absolute values.

### Air pollution–related mortality

The present findings averaged over seven urban centers are similar to those of previous air pollution studies in Chile. In 1989 and 1991, cardiac and respiratory mortality were higher on days of increased PM_10_ (Ostro et al. 1996). Ostro et al. (1996) reported that a 10-μg/m^3^ change in daily mean PM_10_ was associated with a 1% increase in total daily mortality. During the same period, total mortality was associated with PM_2.5_ (Salinas and Vega 1995). Between 1988 and 1996, nonaccidental deaths increased on days of higher air pollution (Cifuentes et al. 2000). Cifuentes et al. (2000) reported that changes in mean levels of pollutants were related to 4–11% changes in mortality. In the present study we found that a change in 24-hr mean PM_10_ of 10 μg/m^3^ was associated with a 1% mortality change, using a single-day lag. This effect occurred largely in the colder months when particulate concentrations were higher, with no statistically significant findings in the warmer months. After adjusting for PM_10_, we also detected an effect of pollutant gases O_3_, CO, and SO_2_ on mortality. An increase in air pollution was associated with an approximate 50% relative increase in respiratory compared to cardiac deaths for CO, SO_2_, and PM_10_.

### Seasonal influences

The mortality effects of CO, SO_2_, and PM_10_ appeared greater during April–September, the colder months, although differences were significant for only PM_10_. Ilabaca et al. (1999) also reported a seasonal modification of the PM_2.5_ effect on pediatric emergency department visits, greatest in the colder months. In the present study, a change in PM_10_ of about 85 μg/m^3^ was associated with a 12.2% change in mortality during the warmer months and 1.3% in the colder months, using unconstrained distributed lags. During the warmer months, October–March, a change in 1-hr maximum daily O_3_ of approximately 100 ppb was associated with a 4.9% change in daily mortality, compared with 2.1% in the colder months. PM_2.5_ is higher in the colder season and O_3_ higher in the warmer season. This could affect the estimate of effect if the exposure–response relation was not linear or if there was a threshold effect. However, the pattern of residuals in our analysis was consistent with a linear effect. Threshold effects have not been documented even at levels of air pollution lower than seen in the present study. Another possible reason for seasonal differences may be different time–activity patterns resulting in different exposures. Finally, there is evidence for seasonal differences in particulate mass composition (crustal vs. combustion sourced) and direction (Celis et al. 2004).

### Effect modification by age

Bell et al. (2005) reported increased mortality effects in the elderly from O_3_ in The National Morbidity, Mortality, and Air Pollution Study of 95 U.S. cities. Bateson and Schwartz (2004) reported that the risk of mortality associated with PM_10_ in Cook County, Ilinois, appeared to increase among elderly women but decreased among elderly men. Filleul et al. (2004) reported a greater effect of air pollution mortality in those > 65 years of age, but it did not reach conventional levels of statistical significance. Others also reported increased susceptibility of those > 65 years of age (Gouveia and Fletcher 2000; Spix et al. 1998). We studied the extremes of old age. Compared with those < 65 years of age, those at least 85 years of age were observed to be over twice as likely to die from acute increases in PM_10_ and > 50% more likely to die from increases in O_3_ and SO_2_. Age-related susceptibility was further magnified when unconstrained distributed lags were considered. We also observed a generally monotonic increase in susceptibility with increasingly older age groups. These findings suggest that the determination of air quality guidelines designed to protect the general population may be insufficient to protect the elderly in our society.

In summary, more recent air pollution data in Santiago, Chile, indicate that air pollution levels continues to be high by comparison with those in North America and are associated with stronger mortality effects from respiratory than cardiac disease. Daily increases in gases and particles are associated with increased mortality. The extremely elderly appear to be at greater risk than those who are younger. We recommend that the degree of susceptibility to air pollution in the very elderly be investigated in other countries to determine whether this finding is generalizable across different climates and air pollution characteristics.

## Figures and Tables

**Table 1:** Population, daily mortality, and ambient air pollution concentrations (ppb) by monitoring location.

						Mean pollutant concentrations		
Region	Population (×10^5^)	Nonaccidental deaths/day	Cardiac deaths/day	Respiratory deaths/day	PM_10_ (μg/m^3^)	O_3_	SO_2_	CO
Las Condes	5.01	5.17	1.61	0.66	65.02	135.52	9.12	0.92
Cerrillos	8.92	11.22	3.01	1.25	84.81	92.94	12.92	1.15
El.Bosque	9.15	11.92	3.15	1.26	86.41	88.02	14.27	1.38
La Florida	13.35	15.52	4.30	1.71	90.22	112.19	13.1	1.43
Independencia	4.21	8.31	2.23	1.10	78.58	89.64	34.06	1.37
Santiago Centro	4.98	8.99	2.83	1.21	82.6	101.72	13.5	1.48
Pudahuel	8.08	8.56	2.08	1.20	91.38	84.94	9.79	1.2
Average	53.70^a^	69.69^a^	19.21^a^	8.38^a^	84.88^b^	100.13^b^	14.08^b^	1.29^b^

a Total number summed over all seven urban centers.

b Population-weighted average pollutant concentration.

**Table 2:** Range of Pearson pairwise correlations between pollutants for seven monitoring locations, 1 January 1997 to 31 December 2003.

Pollutant	PM_10_	O_3_	SO_2_
O_3_	−0.16 to 0.13		
SO_2_	0.37 to 0.77	−0.04 to 0.78	
CO	0.49 to 0.82	−0.55 to −0.01	0.31 to 0.67

**Table 3:** Percent change (t-ratio) in daily nonaccidental mortality associated with changes in pollutant concentrations equivalent to population-weighted averages by individual location.

Location	PM_10_	O_3_	SO_2_	CO
Las Condes	8.37 (1.85)	4.92 (2.31)	9.91 (3.72)	10.58 (3.75)
Cerrillos	11.26 (6.64)	5.80 (3.10)	7.31 (7.30)	5.87 (7.23)
El.Bosque	12.29 (7.33)	6.66 (3.57)	4.84 (5.43)	7.28 (7.96)
La Florida	8.90 (13.08)	3.90 (3.40)	4.15 (4.24)	7.52 (7.92)
Independencia	13.08 (5.42)	2.71 (1.51)	5.98 (5.03)	8.52 (7.89)
Santiago Centro	11.45 (5.83)	5.15 (2.94)	5.48 (5.12)	4.27 (6.06)
Pudahuel	5.77 (3.46)	6.96 (2.66)	6.31 (3.95)	3.48 (4.0)

Single-day lags were used.

**Table 4:** Pooled city estimates of percent change (t-ratio) in daily nonaccidental mortality associated with changes in pollutant concentrations equivalent to population-weighted averages.

Pollutant (mean concentration)	Single-pollutant model	Model adjusted for other pollutants
PM_10_ (84.88 μg/m^3^)	8.54 (5.14)	6.96 (4.02)
O_3_ (100.13 ppb)	5.64 (2.78)	4.13 (2.00)
SO_2_ (14.08 ppb)	5.65 (4.97)	4.51 (3.27)
CO (1.29ppm)	5.88 (6.42)	6.13 (4.34)

Single-day lags were used, and values were adjusted for city-specific temporal trends and weather.

**Table 5:** Percent change (t-ratio) in nonaccidental daily mortality associated with changes in pollutant concentrations equivalent to population-weighted averages by cause of death, age at death, and season.

Classification	PM_10_	O_3_	SO_2_	CO
Cause of death				
Nonaccidental				
Single-day lag	8.54 (5.14)	5.64 (2.78)	5.65 (4.97)	5.88 (6.42)
Distributed lag	11.68 (5.22)	4.38 (2.18)	9.28 (6.64)	9.39 (6.89)
Cardiac				
Single-day lag	10.06 (3.25)	8.78 (2.42)	7.24 (3.55)	7.79 (4.56)
Distributed lag	13.33 (3.35)	2.30 (0.78)	10.53 (4.29)	11.22 (4.8)
Respiratory				
Single-day lag	18.58 (4.51)	8.21 (1.46)	12.45 (4.19)	12.93 (5.78)
Distributed lag	29.66 (4.88)	15.63 (2.50)	20.44 (5.21)	21.31 (6.34)
Age at death (years)				
≤ 64				
Single-day lag	4.53 (1.52)	4.96 (1.17)	4.77 (2.50)	4.10 (2.52)
Distributed lag	4.26 (1.29)	1.84 (0.71)	4.27 (2.49)	4.76 (2.19)
65–74				
Single-day lag	9.47 (2.81)	8.00 (1.77)	5.99 (2.49)	6.24 (3.17)
Distributed lag	11.72 (3.01)	2.15 (0.86)	7.21 (2.55)	8.12 (3.88)
75–84				
Single-day lag	12.61 (3.80)	9.42 (2.28)	8.73 (4.00)	8.64 (4.82)
Distributed lag	17.62 (3.72)	3.32 (0.92)	11.2 (4.25)	13.12 (5.12)
≥ 85				
Single-day lag	14.03 (3.87)	8.56 (2.02)	7.92 (3.23)	8.58 (4.45)
Distributed lag	19.73 (3.75)	5.92 (1.92)	11.13 (4.38)	13.20 (4.82)
Season				
April–September				
Single-day lag	9.12 (3.35)	3.21 (1.14)	6.47 (3.92)	7.09 (4.02)
Distributed lag	12.20 (3.75)	2.14 (1.25)	10.23 (4.72)	9.65 (4.50)
October–March				
Single-day lag	0.60 (0.45)	6.19 (1.92)	2.62 (1.19)	5.45 (1.14)
Distributed lag	1.27 (1.46)	4.89 (1.82)	4.25 (1.75)	7.80 (1.89)
